# Comparative study between histochemical mucus volume, histopathological findings, and endocytoscopic scores in patients with ulcerative colitis

**DOI:** 10.1097/MD.0000000000033033

**Published:** 2023-03-03

**Authors:** Yu Kamitani, Hiroki Kurumi, Tsutomu Kanda, Yuichiro Ikebuchi, Akira Yoshida, Koichiro Kawaguchi, Kazuo Yashima, Yoshihisa Umekita, Hajime Isomoto

**Affiliations:** a Division of Gastroenterology and Nephrology, Department of Multidisciplinary Internal Medicine, Faculty of Medicine, Tottori University, Tottori, Japan; b Department of Pathology, Faculty of Medicine, Tottori University, Tottori, Japan.

**Keywords:** endocytoscopy, mucus volume, ulcerative colitis

## Abstract

Ulcerative colitis (UC) causes a reduction in goblet cells. However, there have been few reports on the relationship between endoscopic and pathological findings and mucus volume. In this study, we quantitatively evaluated histochemical colonic mucus volume by fixing biopsied tissue sections taken from patients with UC in Carnoy’s solution and compared it with endoscopic and pathological findings to determine whether there is a correlation between them. Observational study. A single-center, university hospital in Japan. Twenty-seven patients with UC (male/female, 16/11; mean age, 48.4 years; disease median duration, 9 years) were included in the study. The colonic mucosa of the most inflamed area and the surrounding less inflamed area were evaluated separately by local MES and endocytoscopic (EC) classification. Two biopsies were taken from each area; one was fixed with formalin for histopathological evaluation, and the other was fixed with Carnoy’s solution for the quantitative evaluation of mucus via histochemical Periodic Acid Schiff and Alcian Blue staining. The relative mucus volume was significantly reduced in the local MES 1–3 groups, with worsening findings in EC-A/B/C and in groups with severe mucosal inflammation, crypt abscess, and severe reduction in goblet cells. The severity of inflammatory findings in UC by EC classification correlated with the relative mucus volume suggesting functional mucosal healing. We found a correlation between the colonic mucus volume and endoscopic and histopathological findings in patients with UC, and a stepwise correlation with disease severity, particularly in EC classification.

## 1. Introduction

Ulcerative colitis (UC) is histopathologically characterized by the depletion of goblet cells, which produce and secrete mucin, the main component of the mucosa.^[1–5]^ The mucus released by goblet cells in the intestine is mainly composed of glycoproteins, such as MUC2, and the mucus layer is divided into the outer and inner mucus layers.^[6,7]^ The maintenance of such a mucus layer serves as a physical primary barrier that prevents easy contact between intestinal microflora, which are abundant in the gastrointestinal tract lumen, and epithelial cells.^[8,9]^ Inflammatory bowel diseases, including UC, disrupt intestinal barrier functions, such as the mucus layer and antimicrobial peptides.^[10]^ In MUC2 knockout mice, the inner mucus layer (a major component of mucus) was not formed in the large intestine, and invasion of bacteria into the intestinal epithelial tissues and spontaneous onset of inflammation were observed.^[11]^ Such reports suggest the importance of mucus in the intestinal tract.

The relationship between disease activity and changes in mucus volume in UC has been studied. Nakano et al^[12]^ studied the correlation between goblet cell depletion and quantitative changes in mucin using a rat UC model and found localized goblet cell depletion and mucin reduction in the colon near the anal portion of the highly inflamed lesion. Nogami et al^[13]^ took mucosal biopsies from the rectum of patients with UC in areas that were almost normal and areas where inflammation or ulceration was observed and examined the morphological characteristics of the rectal mucosa using glycohistochemistry, scanning electron microscopy, and transmission electron microscopy. They surmised that goblet cells are reduced in the crypts of colorectal lesions compared to the surrounding near-normal areas, resulting in reduced mucus discharge from the crypt. Thus, although scattered studies have reported a local reduction in mucus volume in the inflamed area of the colon in UC, there are few reports on humans that have shown whether quantitative changes in mucus are associated with a more clinical disease course. In this study, we quantitatively assessed relative mucin volume via histochemistry with Periodic Acid Schiff (PAS) and Alcian Blue (AB) staining using tissue biopsy sections taken from patients with UC that were fixed with Carnoy’s solution and compared them with endoscopic findings, including ultra-high magnification, and pathological findings. The objective of this study was to investigate the correlation between endoscopic and histopathologic disease activity and colonic mucus volume in patients with ulcerative colitis.

## 2. Methods

**Figure 1.**
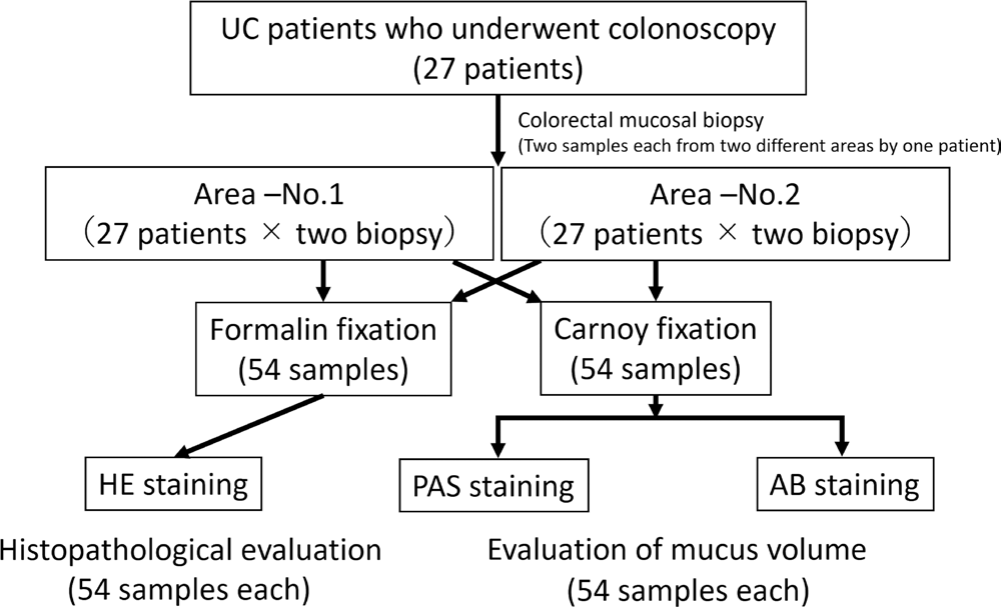
Flow chart from endoscopic biopsy to fixation and staining. AB = Alcian Blue, HE = hematoxylin-eosin, PAS = Periodic Acid Schiff, UC = ulcerative colitis.

This study included 27 Japanese patients (16 men, 11 women) aged ≥20 years (range, 26–76 years; mean, 48.4 years) diagnosed with UC at Tottori University Hospital, Tottori, Japan, who underwent total colonoscopy using ultra-high magnification endoscopy (Endocyto, OLYMPUS GIF TYPE H-290EC, Olympus Corp., Tokyo, Japan) between April 2019 and November 2020. The endoscopic activity in each patient was evaluated using the Mayo endoscopic subscore (MES).^[5]^ The colonic mucosa of the most inflamed area and the surrounding less inflamed area were evaluated separately using MES, which was defined as local MES (L-MES). Endocytoscopic (EC) classification^[14]^ was used to evaluate the microstructural endoscopic activity and to assess mucosal healing in two different mucosal areas in each patient. Two biopsies were obtained from each area. One of the biopsy tissues was subjected to formalin-fixed hematoxylin-eosin (HE) staining for pathological evaluation based on the three categories of characteristic UC findings: severity of mucosal inflammation; presence of crypt abscess; and severity of goblet cell depletion. The second biopsy tissue was subjected to Carnoy-fixed PAS and AB staining to quantitatively assess the histochemical mucus volume in the lumen of the colonic gland duct (Fig. 1).

The clinical data of patients were aggregated and included age, sex, disease duration, disease type classification, Sutherland index (disease activity index; DAI),^[15]^ medications, general laboratory findings, MES, L-MES, EC classification, tissue sampling site, and histopathological findings (severity of mucosal inflammation, presence of crypt abscess, and severity of goblet cell depletion). The study was approved by the Tottori University Hospital (no. 1512A094) and was conducted in accordance with the Helsinki Declaration. Informed consent was obtained from all patients who participated in this study.

### 2.1. Fixation and staining

#### 2.1.1. Formalin fixation and HE staining.

Tissue samples were fixed in 10% neutral buffered formalin for 24 hours and transferred into low-melting-point paraffin. To qualify the material for histochemistry, sections were stained with HE.

#### 2.1.2. Carnoy’s solution fixation.

The composition of Carnoy’s solution was 10% ethanol, chloroform, and glacial acetic acid in a ratio of 6:3:1. The collected tissues were fixed for 2 hours in Carnoy’s solution and placed in 100% ethanol for 2 to 3 hours to remove the acetic acid and chloroform contained in the Carnoy’s solution before embedding.

#### 2.1.3. PAS staining.

**Figure 2.**
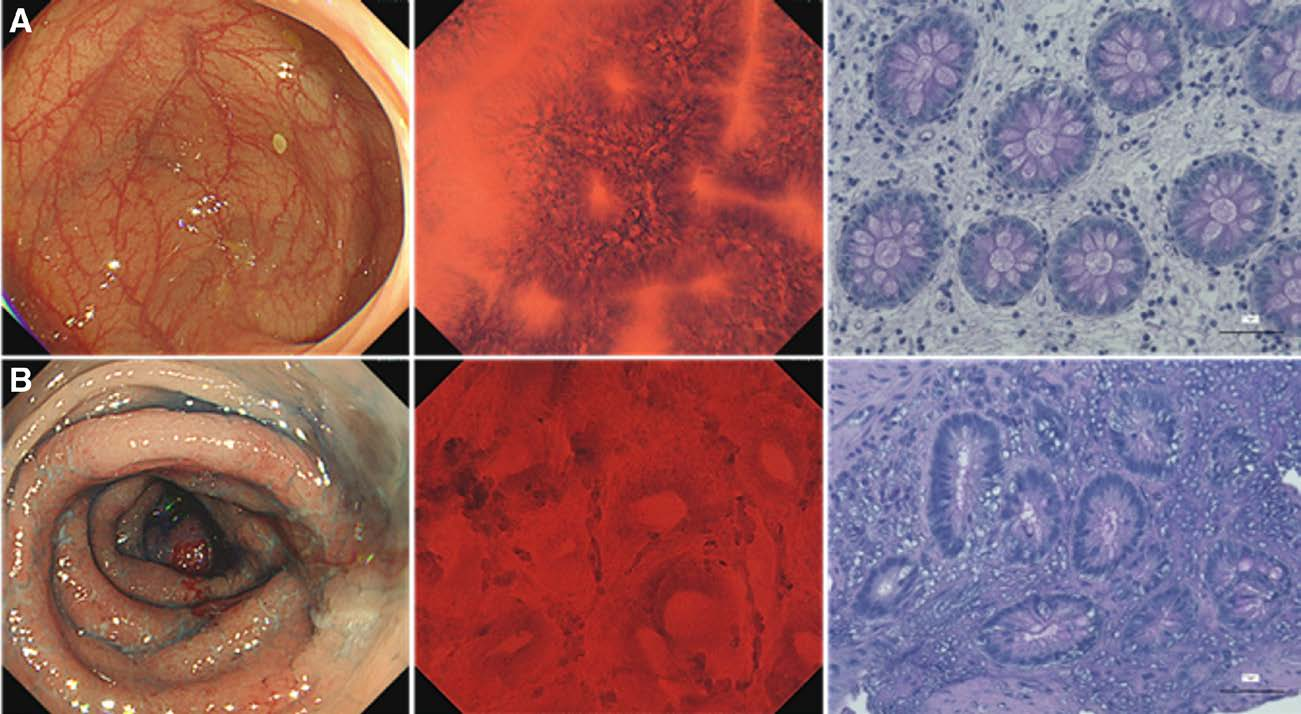
Histochemical image of colonic gland stained with PAS in the same patient. (A) Typical L-MES 0 and EC-B images and PAS-stained image of the biopsy specimen from the same site. (B) Typical L-MES 2 and EC-C images and PAS-stained image of the biopsy specimen from the same site. EC = endocytoscopic classification, L-MES = local mayo endoscopic subscore, PAS = Periodic Acid Schiff.

To qualitatively detect neutral mucin polysaccharides in epithelial mucin/mucosal material, PAS reaction with Schiff’s reagent was applied (Fig. 2). Sections on microscope slides were deparaffinized (using a non-xylene alternative), rinsed with water, and passed through distilled water; immersed in 1% periodic acid solution (1% HIO_4_ 2H_2_O) for 10 minutes, rinsed with water for 1 minutes, and passed through distilled water three times; samples were immersed in Schiff’s reagent for 30 minutes as a color reaction (reddish-purple), washed three times with 0.5% sodium disulfite for 3 minutes, washed with running water for 5 minutes, and washed with distilled water; for counterstaining of the cell nucleus (purple-blue color), cells were soaked in Mayer’s hematoxylin solution for 2 minutes, rinsed with running water for 2 minutes, and rinsed with warm water for 5 minutes to remove the color; slides were dehydrated by ascending alcohol series; penetrated using alternative xylene; and sealed using a non-xylene sealant.

#### 2.1.4. AB staining.

**Figure 3.**
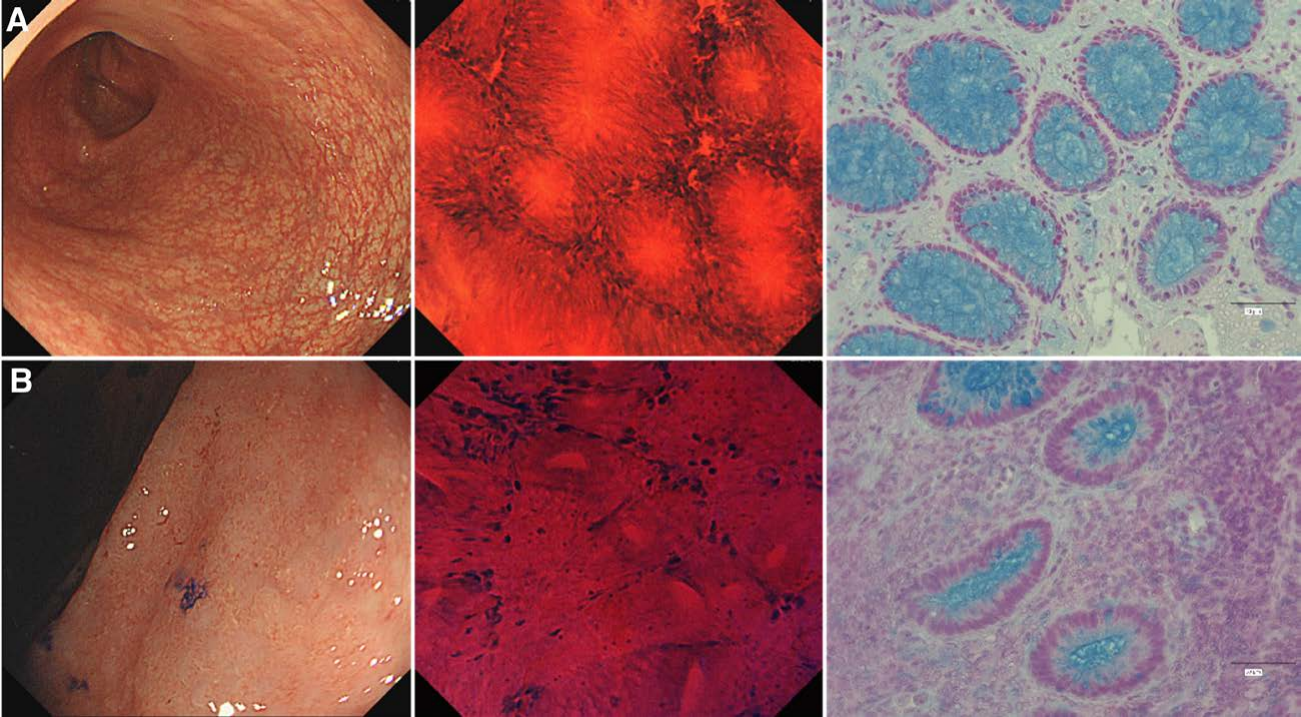
Histochemical image of colon gland stained with AB in the same patient. (A) Typical L-MES 0 and EC-B images and AB stained image of the biopsy specimen from the same site. (B) Typical L-MES 2 and EC-C images and AB stained image of the biopsy specimen from the same site. AB = Alcian Blue, EC = endocytoscopic classification, L-MES = local mayo endoscopic subscore.

To selectively detect epithelial acid mucins, non-sulfated mucins (sialomucins: dark blue), and sulfated acid mucins (sulfomucins: light blue), AB staining at pH 2.5 was performed (Fig. 3). Slides were deparaffinized and hydrated with distilled water; immersed in 3% acetic acid (pH 2.5) for 3 minutes and AB solution (pH 2.5) for 30 minutes, rinsed with 3% acetic acid water (pH 2.5) to remove excess AB, and rinsed under running water; for nuclear staining (red), the slides were immersed in Kernechtrot solution for 5 minutes and washed for 2 minutes in running water; dehydrated by ascending alcohol series; penetrated using alternative xylene; and sealed using a non-xylene sealant.

### 2.2. Calculation method of relative colonic mucus volume area

**Figure 4.**
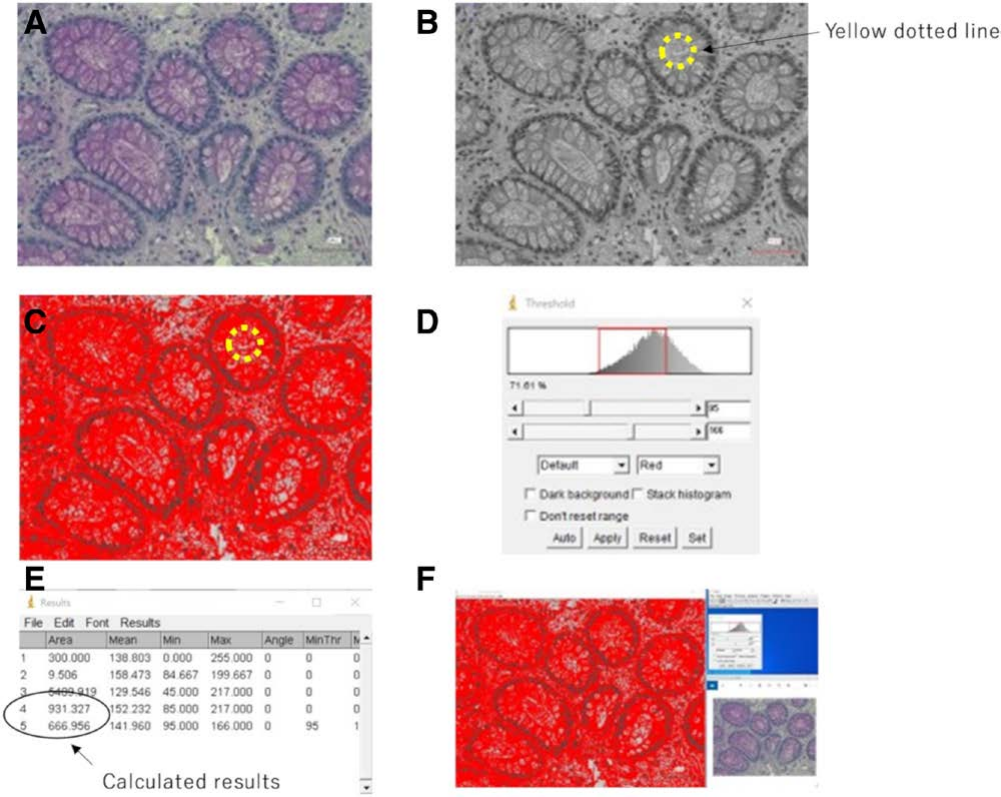
Process of calculating the area of gland ducts using ImageJ. (A) Original pathological images of Carnoy’s-fixed PAS staining. (B) Binarize a color image and surround the lumen of the gland duct with a yellow dotted line. (C) Adjust the threshold to match the area occupied by mucus in the lumen of the gland duct and calculate only the red-colored area of the lumen. (D) The adjusted threshold. (E) The calculated area of the lumen is shown in No. 4 of the results, and the area of the glandular lumen surrounded in red (area of mucus) is shown in No. 5. (F) Overall image. PAS = Periodic Acid Schiff.

Glass slides stained with Carnoy-fixed PAS and AB stains were observed under a 40 × objective lens of a microscope (BZ-X710; KEYENCE Corp., Osaka, Japan) linked to a digital camera. Ten gland ducts were randomly allocated and saved as images (1920 × 1440 pixels in size). ImageJ 1.53e (Wayne Rasband and contributors, National Institutes of Health, Madison, WI) was used to binarize the color image and perform threshold extraction. The threshold value was adjusted to match the area occupied by the mucus in the lumen of the gland duct, calculate the area of the gland duct lumen (Q μm^2^), the area of the mucus inside it (P μm^2^), and define the ratio P/Q. This procedure was performed for each of the 54 PAS and AB staining samples for a total of 1080 gland ducts. Figure 4 shows the process of calculating the area of a pathological image.

### 2.3. Statistical analysis

All results were tested using the Kolmogorov–Smirnov test. The results showed a lack of normality; therefore, a nonparametric approach was used for the analysis. Continuous variables were expressed as mean ± standard deviation or median with interquartile range, according to the distribution. Kruskal–Wallis test was performed on three or more unrelated groups for the differences in the relative glycohistochemical mucus volume for each L-MES group (L-MES 0/1/2/3) and each EC classification group (EC-A/B/C/D) for each sample. Dunn test and Bonferroni adjustment were then performed for each of the two groups in multiple comparisons. The results were also validated using the Mann–Whitney *U* test between the two groups without correspondence for the severity of mucosal inflammation (none to mild/moderate to severe), presence of crypt abscess, and severity of goblet cell reduction (none to mild/moderate to severe). IBM SPSS version 27.0 (IBM Corp., Armonk, NY) was used for statistical analysis, and statistical significance was set at *P *< .05.

### 2.4. Patient and public involvement

This study is an observational study. The patients with UC attending the Department of Gastroenterology at Tottori University Hospital, who gave written consent to participate in the study, are included. Patients were not directly involved in the study design. The results of the study will be published as a contributed article after the study is completed.

## 3. Results

### 3.1. Patient characteristics

Total colonoscopy for microstructural assessment of EC classification was performed in 27 patients with UC. There were 16 male patients and 11 female patients. The median duration of UC in these patients was 8.93 years. The disease types were pancolitis in 14 patients, left-sided colitis in seven patients, and proctitis in six patients. The median value of the Sutherland index (DAI) was 1. At the time of endoscopy, 13 patients were treated with 5-aminosalicylic acid (5-ASA) alone, two were treated with 5-ASA + PSL (prednisolone) orally, two were treated with 5-ASA + topical PSL, seven were treated with 5-ASA + IM (immunomodulator), one was treated with 5-ASA + topical PSL + IM, one was treated with 5-ASA + IM + biologics (Bio), and one was treated with PSL orally + IM. Major laboratory findings included a median white blood cell count of 5500 × 10^3^/µL (range, 3500–111,200), median C-reactive protein of 0.04 mg/dL (range, 0.01–0.22), and median erythrocyte sedimentation rate of 12 mm/h (range, 1–44). The MES details were as follows: MES 0, 10 cases; MES 1, seven cases; MES 2, nine cases; and MES 3, one case. The L-MES was distributed as follows: L-MES 0, 37; L-MES 1, seven; L-MES 2, nine; L-MES 3, one. The EC classification was distributed as follows: EC-A, 31; EC-B, 15; EC-C, eight; EC-D, 0. The biopsy location was the ileocecum in three cases, ascending colon in eight cases, transverse colon in four cases, descending colon in seven cases, sigmoid colon in seven cases, and rectum in 25 cases. The histopathological findings were as follows: the severity of mucosal inflammation was none-mild in 36 cases and moderate-severe in 18 cases. Crypt abscesses were absent in 49 cases and present in five cases. The severity of goblet cell depletion was none-mild in 48 cases and moderate-severe in six cases (Table 1).

**Table 1:** Clinical characteristics of 27 patients at the time of endoscopic observation.

Characteristics	n = 27
Age, years, mean (SD)	48.4 ± 15.5
Sex, male/female	16/ 11
Duration of disease, yr, median (IQR)	8.93 (4.25–13.0)
Location of disease, n (%)	
Pancolitis	14 (51.8)
Left-side colitis	7 (25.9)
Proctitis	6 (22.2)
DAI, median (IQR)	1 (0–3)
Treatment, n (%)	
5-ASA	13 (48.1)
5-ASA + oral PSL	2 (7.4)
5-ASA + Topical PSL	2 (7.4)
5-ASA + IM	7 (25.9)
5-ASA + IM + Topical PSL	1 (3.7)
5-ASA + IM + Bio	1 (3.7)
Oral PSL + IM	1 (3.7)
Laboratory data, median (IQR)	
WBC/µL	5500 (3500–11200)
CRP, mg/L	0.04 (0.01–0.22)
ESR, mm	12 (1–44)
MES, n (%)	
0	10 (37.0)
1	7 (25.9)
2	9 (33.3)
3	1 (3.7)
L-MES, n (%)	
0	37 (68.5)
1	7 (12.9)
2	9 (16.7)
3	1 (1.8)
EC classification, n (%)	
A	31 (57.4)
B	15 (27.7)
C	8 (14.8)
D	0 (0.0)
Location of biopsy tissues, n (%)	
Ileocecum	3 (5.6)
Ascending colon	8 (14.8)
Transverse colon	4 (7.4)
Descending colon	7 (12.9)
Sigmoid colon	7 (12.9)
Rectum	25 (46.3)
Severity of mucosal inflammation, n (%)	
None-mild	36 (66.6)
Moderate-severe	18 (33.3)
Crypt abscesses, n (%)	
Absence	49 (90.7)
Presence	5 (9.2)
Severity of goblet cell depletion, n (%)	
None-mild	48 (88.8)
Moderate-severe	6 (11.1)

5-ASA = 5-aminosalicylic acid, Bio = biologics, CRP = C-reactive protein, DAI = disease activity index, EC = endocytoscopic, ESR = erythrocyte sedimentation rate, IM = immunomodulator, IQR = interquartile range, L-MES = local mayo endoscopic subscore, MES = mayo endoscopic subscore, PSL = prednisolone, SD = standard deviation, WBC = white blood cell.

### 3.2. Comparison of histochemical mucus volume between L-MES groups

In PAS staining, the mean P/Q ratio was 0.58, 0.48, 0.45, and 0.33 for L-MES 0, 1, 2, and 3, respectively. The mucus volume was significantly reduced in the two-group comparison between each group of L-MES 1–3 when compared to L-MES 0 (*P* < .001). The mucus volume was significantly reduced in L-MES 3 compared to L-MES 1 (*P* = .048), though there was no significant difference between L-MES 1 and L-MES 2 or between L-MES 2 and L-MES 3 (*P* = 1.000 and *P* = .253, respectively).

**Figure 5.**
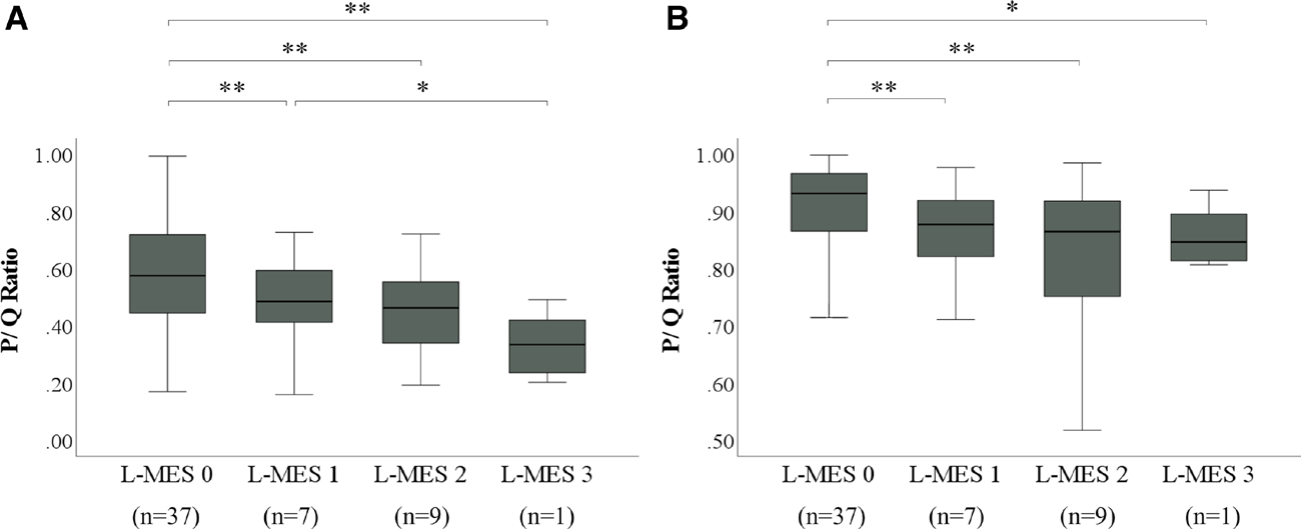
Correlation between L-MES and mucus volume. (A) Comparison of histochemical mucus volume among local L-MES in PAS staining. (B) Comparison of histochemical mucus volume among local L-MES in AB staining. The length of the box represents the interquartile range. The line through the middle of each box represents the median. Bars indicate the minimum and maximum values. *P = .048 (Dann–Bonferroni); **P < .001 (Dann–Bonferroni). AB = Alcian Blue, L-MES = local mayo endoscopic subscore, P = area of mucus in the lumen of the gland duct, PAS = Periodic Acid Schiff, Q = area of the lumen of the gland duct.

In AB staining, the mean P/Q ratio was 0.90, 0.85, 0.79, and 0.85 for L-MES 0, 1, 2, and 3, respectively. There was a significant reduction in the mucus volume in the two-group comparison between each group of L-MES 1–3 compared to L-MES 0 (*P* < .001 between L-MES 0 and L-MES 1 and between L-MES 0 and L-MES 2; *P* = .048 between L-MES 0 and L-MES 3). However, there was no significant difference in the two-group comparison between each of the L-MES 1–3 groups (*P* = 1.000, respectively) (Fig. 5).

### 3.3. Comparison of histochemical mucus volume between EC classification groups

In PAS staining, the mean P/Q ratio was 0.58, 0.51, and 0.43 for EC-A, B, and C, respectively. The mucus volume was significantly reduced in EC-B and EC-C compared to EC-A (*P* < .001 and *P* = .009, respectively). There was also a significant reduction in mucus volume in EC-C compared to EC-B (*P* < .001).

**Figure 6.**
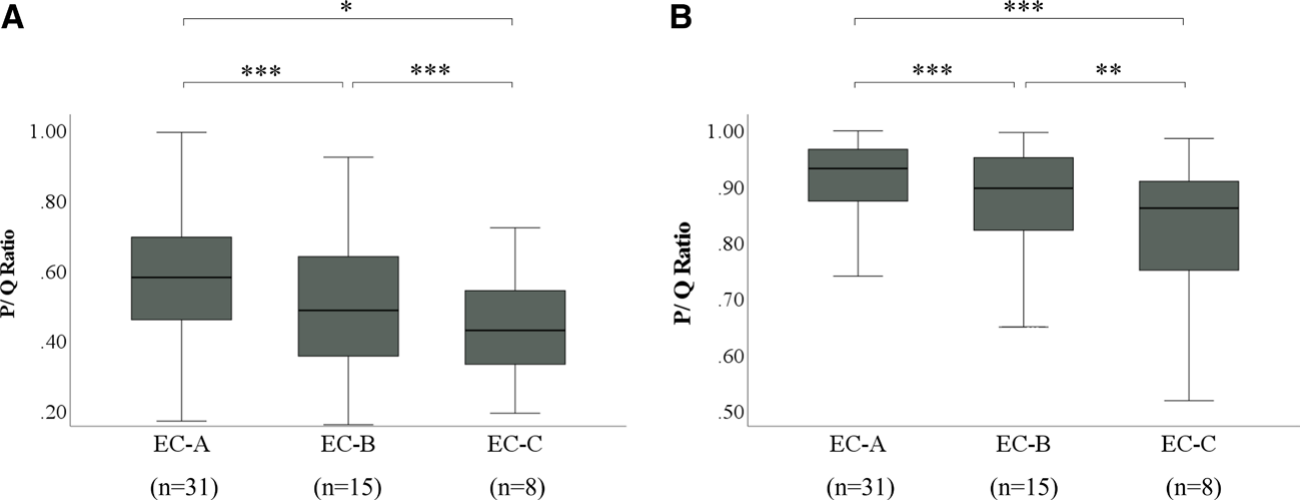
Correlation between EC and mucus volume. (A) Comparison of histochemical mucus volume among EC in PAS staining. (B) Comparison of histochemical mucus volume among EC in AB staining. The length of the box represents the interquartile range. The line through the middle of each box represents the median. Bars indicate the minimum and maximum values. *P = .009 (Dann–Bonferroni); **P = .002 (Dann–Bonferroni); ***P < .001 (Dann–Bonferroni). AB = Alcian Blue, EC = endocytoscopic classification, P = area of mucus in the lumen of the gland duct, PAS = Periodic Acid Schiff, Q = area of the lumen of the gland duct.

In AB staining, the mean P/Q ratio was 0.90, 0.87, and 0.78 for EC-A, B, and C, respectively. The mucus volume was significantly reduced in EC-B and EC-C compared to EC-A (*P* < .001). There was also a significant reduction in the mucus volume in EC-C compared to EC-B (*P* = .002) (Fig. 6).

### 3.4. Comparison by the severity of mucosal inflammation

In PAS staining, the mean ratio P/Q according to the severity of mucosal inflammation was 0.56 and 0.50 for non-mild and moderate-to-severe inflammation, respectively. The mucus volume was significantly reduced in the moderate-to-severe group compared to the non-mild group (*P* < .001).

**Figure 7.**
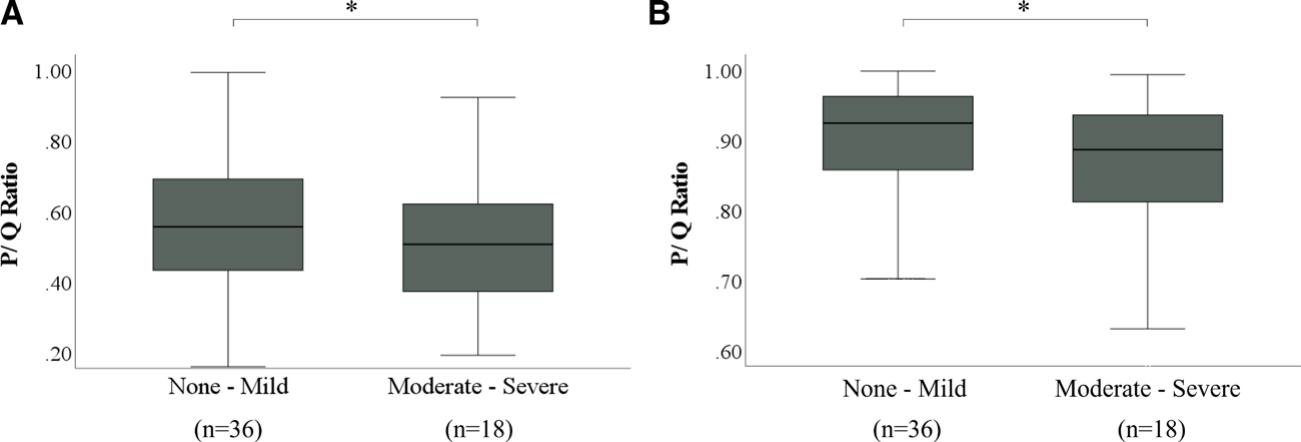
Correlation between severity of mucosal inflammation and mucus volume. (A) Comparison of histochemical mucus volume according to the severity of mucosal inflammation in PAS staining. (B) Comparison of histochemical mucus volume according to the severity of mucosal inflammation in AB staining. The length of the box represents the interquartile range. The line through the middle of each box represents the median. Bars indicate the minimum and maximum values. *P < .001 (Mann–Whitney *U* test). AB = Alcian Blue, P = area of mucus in the lumen of the gland duct, PAS = Periodic Acid Schiff, Q = area of the lumen of the gland duct.

In AB staining, the mean ratio P/Q according to the severity of mucosal inflammation was 0.89 and 0.83 for non-mild and moderate-to-severe inflammation, respectively. The mucus volume was significantly reduced in the moderate-to-severe group compared to the non-mild group (*P* < .001) (Fig. 7).

### 3.5. Comparison by the presence of crypt abscess

In PAS staining, the mean P/Q ratio was 0.55 and 0.45 in the group without and with crypt abscess, respectively. The mucus volume was significantly reduced in the present group compared to the absent group (*P* < .001).

**Figure 8.**
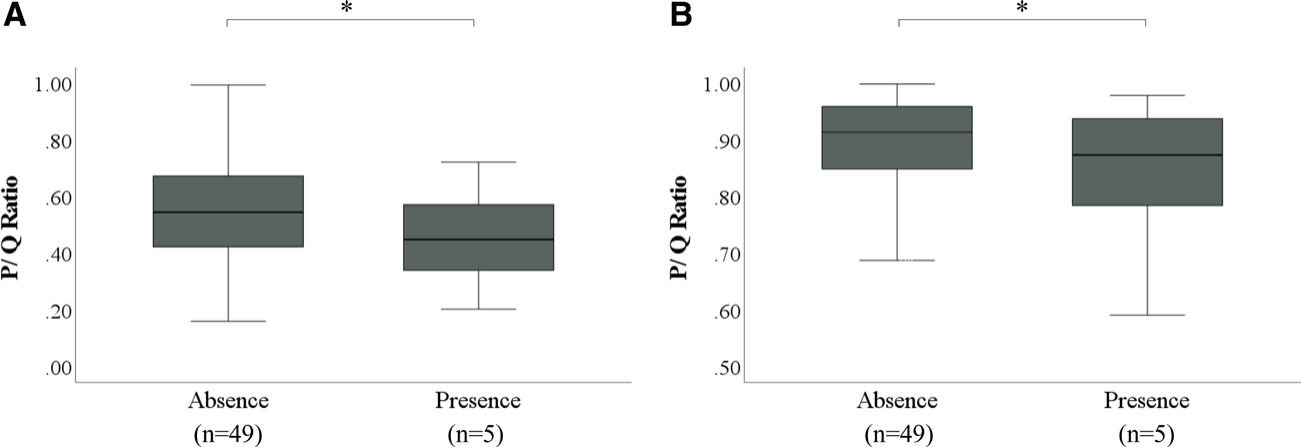
Correlation between the presence of crypt abscess and mucus volume. (A) Comparison of histochemical mucus volume according to the presence of crypt abscess in PAS staining. (B) Comparison of histochemical mucus volume according to the presence of crypt abscess in AB staining. The length of the box represents the interquartile range. The line through the middle of each box represents the median. Bars indicate the minimum and maximum values. *P < .001 (Mann–Whitney *U* test). AB = Alcian Blue, P = area of mucus in the lumen of the gland duct, PAS = Periodic Acid Schiff, Q = area of the lumen of the gland duct.

In AB staining, the mean P/Q ratio was 0.88 and 0.81 in the group without and with crypt abscess, respectively. The mucus volume was significantly reduced in the present group compared to the absent group (*P* < .002) (Fig. 8).

### 3.6. Comparison by the severity of goblet cell depletion

In PAS staining, the mean P/Q ratio was 0.55 and 0.48 in the non-mild and moderate-to-severe goblet cell depletion groups, respectively. The mucus volume was significantly reduced in the moderate-to-severe group compared to the non-mild group (*P* < .005).

**Figure 9.**
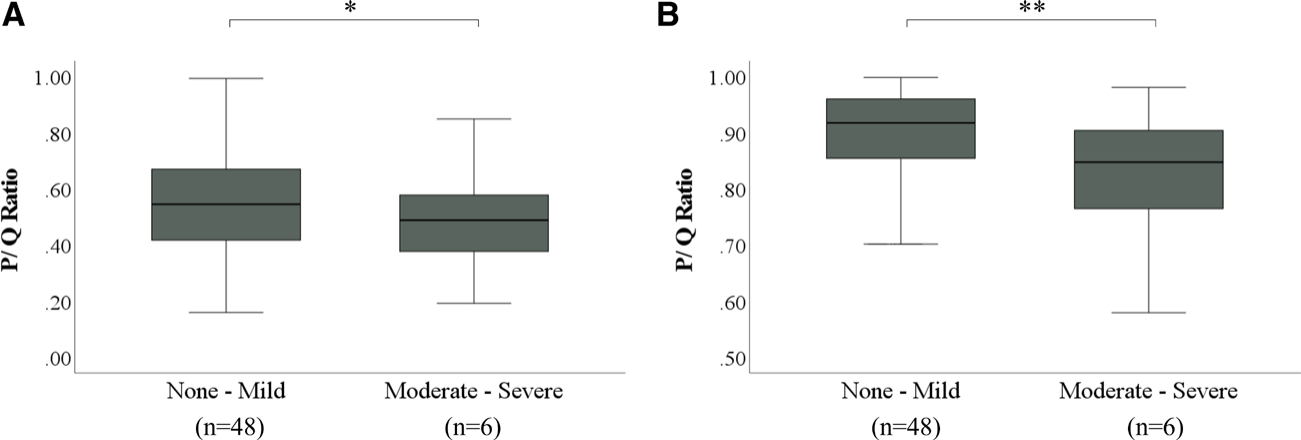
Correlation between the severity of goblet cell depletion and mucus volume. (A) Comparison of histochemical mucus volume according to the severity of goblet cell depletion in PAS staining. (B) Comparison of histochemical mucus volume according to the severity of goblet cell depletion in AB staining. The length of the box represents the interquartile range. The line through the middle of each box represents the median. Bars indicate the minimum and maximum values. *P = .005 (Mann–Whitney *U* test). **P < .001 (Mann–Whitney *U* test). AB = Alcian Blue, P = area of mucus in the lumen of the gland duct, PAS = Periodic Acid Schiff, Q = area of the lumen of the gland duct.

In AB staining, the mean P/Q ratio was 0.88 and 0.81 in the non-mild and moderate-to-severe goblet cell depletion groups, respectively. The mucus volume was significantly reduced in the moderate-to-severe group compared to the non-mild group (*P* < .001) (Fig. 9).

## 4. Discussion

In this study, we quantitatively evaluated the relative colonic mucus volume assessed by PAS and AB staining using Carnoy-fixed tissue sections of colorectal mucosal biopsies taken from patients with UC and correlated them with endoscopic activity, EC classification, and histopathological findings in UC. Takada et al^[16]^ reported in murine cecal mucosa, and Blick et al^[17]^ reported in porcine intestine that Carnoy’s solution fixation is more suitable than formalin fixation in terms of preservation of the intestinal mucus layer. Swidsinski et al^[18,19]^ reported preserving the intestinal mucus layer in the human colon in inflammatory bowel disease (IBD) using Carnoy’s solution fixation, which facilitated the detection of bacteria adhering to the surface. Based on these previous studies, we evaluated the mucus volume using specimens fixed in Carnoy’s solution and histopathological findings using specimens fixed in formalin solution.

McCormick et al^[20]^ reported that there was a significant reduction in the amount of mucin in patients with UC who underwent rectal biopsy compared to those with Crohn’s disease and healthy controls. Furthermore, Wu et al^[21]^ reported the usefulness of moxibustion treatment in patients with active UC and the changes in the amount of mucin before and after treatment. The study showed that most patients had improved endoscopic inflammatory findings, such as redness and edema, and histopathological findings, such as reduced inflammatory cell infiltration and absence of crypt abscesses. In addition, the amount of mucus mucin that was reduced before treatment was improved after treatment. In the present study, there was also a significant correlation between the endoscopic and histopathological activities of UC and mucus volume. Specifically, there was significantly more mucus remaining in areas where mucosal healing was achieved than in areas where mucosal healing was not achieved. In addition, there was significantly more mucus remaining in areas where histological remission was achieved than in areas where histological remission was not achieved, which was consistent with previous reports.^[21]^

There is a report showing that some patients have an almost normal mucosa with no inflammation endoscopically, yet histopathological evaluation shows residual active inflammatory cell infiltration. Persistent histopathological inflammation has been associated with a 2- to 3-fold increased risk of colitis relapse, corticosteroid use, and hospitalization over the next 12 months.^[22]^ In recent years, achieving mucosal healing in addition to histological remission in UC has been shown to lower the risk of clinical relapse,^[23–26]^ which has become a common treatment goal. However, biopsy evaluation is not a real-time evaluation and is costly and time-taking.^[27]^ Moreover, there is a risk of tissue damage such as bleeding.^[28–30]^ We previously proposed EC classification using ultra-high magnification endoscopy and reported that it correlated significantly with histopathological findings in patients with UC.^[14]^ Accordingly, the current study employed the EC classification stratified into four categories: EC-A, regular arrangement of round-to-oval pits; EC-B, irregular arrangement with/without enlarged spaces between regular pits; EC-C, deformed pits with distorted crypt lumen that are unordered in arrangement but not disrupted; and EC-D, disruptive or disappeared pits. We also reported that EC classification correlated with the relapse rate and that achieving EC-A reduced the relapse rate during the long-term follow-up.^[14]^

According to the current expert consensus, complete endoscopic healing is defined as MES-0.^[31]^ However, no less than 10% or more of patients with MES-0 show clinical relapse within 12 months.^[32]^ More recently, Takishima et al^[33]^ demonstrated that the on-site observation of goblet appearance on endocytoscopy could predict future prognosis in UC patients with MES-0, since the depleted-goblet group had a significantly higher cumulative clinical relapse rate than the preserved-goblet group (19% [15/81] vs 5% [2/39], respectively; *P* = .02). In support of this, histologic goblet mucin depletion has emerged as a predictor of clinical relapse in patients with MES-0.^[34]^ Nevertheless, manually counting the number of goblet appearances in the study was a time-consuming approach and a facet of weakness for the approach in clinical practice. In the present study, there was a significant correlation between EC classification and the relative mucus volume, and the mucus volume showed a stepwise reduction with the worsening of EC classification findings. This suggests that recovery of mucin production may have been achieved in particular patients with UC classified as EC-A because the ultrastructure was sufficiently restored accompanied by round-to-oval pits. Improvement in the amount of intestinal mucus is thought to reflect functional healing, and future studies are needed to examine the relationship between functional healing, relapse rates, and carcinogenesis.

The limitation of this study was its single-centered design with a relatively small number of cohorts. Second, the distribution of patients with UC was weighted toward those with relatively mild phase MES 0-1, which may have resulted in a lack of significant stepwise differences in the mucus volume by L-MES severity. However, when performing ultra-high magnifying observation with endocytoscopy, it is difficult to visualize glandular structures and cell nuclei on mucosal surfaces with spontaneous bleeding or ulceration due to the higher degree of inflammation, making it difficult to evaluate EC classification. Therefore, it can be said that the group of patients with relatively mild activity, which is the main target of this study, could be appropriate. Meanwhile, the strength of the EC system is good compatibility with artificial intelligence.^[35]^ The development of artificial intelligence-assisted EC classification in UC, especially categorized as MES-0, may replace the current, somewhat subjective judgment, depending on the attending endoscopists with expertise still lacking common agreement worldwide.

In conclusion, we found a correlation between the colorectal mucus volume and endoscopic and histopathological findings in patients with UC, and a stepwise correlation with disease severity, particularly in EC classification.

## Acknowledgments

We would like to thank Editage (www.editage.com) for English language editing. We would like to thank the staff of the department of pathology, Faculty of Medicine, Tottori university for their cooperation in histopathological diagnosis.

## Author contributions

**Conceptualization:** Yu Kamitani, Hiroki Kurumi, Hajime Isomoto.

**Data curation:** Yu Kamitani, Hiroki Kurumi, Hajime Isomoto.

**Formal analysis:** Yu Kamitani, Hiroki Kurumi, Hajime Isomoto.

**Investigation:** Yu Kamitani, Hiroki Kurumi, Tsutomu Kanda, Hajime Isomoto.

**Methodology:** Yu Kamitani, Hiroki Kurumi, Tsutomu Kanda, Hajime Isomoto.

**Resources:** Yu Kamitani, Hiroki Kurumi, Yuichiro Ikebuchi, Akira Yoshida, Koichiro Kawaguchi, Kazuo Yashima, Yoshihisa Umekita, Hajime Isomoto.

**Supervision:** Hajime Isomoto.

**Validation:** Yu Kamitani, Hiroki Kurumi, Hajime Isomoto.

**Writing – original draft:** Yu Kamitani.

**Writing – review & editing:** Hiroki Kurumi, Hajime Isomoto.
